# Lupenone-Rich Fraction Derived from *Cissus quadrangularis* L. Suppresses Lipid Accumulation in 3T3-L1 Adipocytes

**DOI:** 10.3390/life13081724

**Published:** 2023-08-11

**Authors:** Thitiporn Lakthan, Panupun Limpachayaporn, Kanok-on Rayanil, Pornsri Charoenpanich, Pornwipa Phuangbubpha, Adisri Charoenpanich

**Affiliations:** 1 Department of Biology, Faculty of Science, Silpakorn University, Nakhon Pathom 73000, Thailand; lakthan_t@silpakorn.edu (T.L.); phuangbubpha_p@silpakorn.edu (P.P.); 2 Department of Chemistry, Faculty of Science, Silpakorn University, Nakhon Pathom 73000, Thailand; limpachayaporn_p@su.ac.th (P.L.); rayanil_k@su.ac.th (K.-o.R.); 3 Department of Food Technology, Faculty of Engineering and Industrial Technology, Silpakorn University, Nakhon Pathom 73000, Thailand; charoenpanich_p@su.ac.th

**Keywords:** *Cissus quadrangularis*, 3T3-L1, antiadipogenesis, hexane extract, lupenone, glucose uptake, mitochondrial density, *Pparg2*

## Abstract

*Cissus quadrangularis* L. (CQ) has potential as a therapeutic for managing obesity and balancing metabolic activity, but the main bioactive compound and regulatory mechanism remain unknown. Herein, the CQ hexane extract was fractionated into 30 fractions (CQ-H) using flash column chromatography and analyzed using thin-layer chromatography. The direct antiadipogenesis effect of CQ-H fractions was tested on 3T3-L1 preadipocytes. Lupenone-rich fractions 2H and 3H were identified as containing potent antiadipogenesis agents that reduced differentiated cell numbers and intracellular lipid droplet size. Although the overall mitochondrial density remained unchanged, differentiated cells exhibited a higher mitochondrial density than that in non-differentiated cells. Additionally, 2H increased mitochondrial activity in both cell types as shown by their differentiation and lipid formation stages. Lupenone was isolated from 2H (Lu-CQ) and shown to dose-dependently inhibit adipogenesis, with 2H being more potent than Lu-CQ. Lu-CQ and 2H downregulated the expression of *Pparg2* mRNA and upregulated that of glucose transporter genes, *Slc2a1* and *Slc2a4*. Lu-CQ and 2H induced increased glucose uptake by 3T3-L1 cells. These findings suggest that lupenone-rich fractions in CQ contribute to balancing metabolic activity and reducing adipose tissue formation. Further exploration of CQ and its components may prompt innovative strategies for managing obesity and metabolic disorders.

## 1. Introduction

Adipose tissue plays a crucial role as an insulator, mechanical absorber, and energy storage and helps regulate metabolic activity. An imbalance in adipose tissue formation and adipocyte function can produce various disorders and health complications [1]. The excessive accumulation of adipose tissue, commonly observed in overweight and obesity, is linked to an increased risk of cardiovascular diseases, type 2 diabetes, and certain types of cancer [2]. Furthermore, the functional integrity of adipose tissue is of utmost importance as this influences insulin sensitivity, blood glucose levels, inflammation, and lipid metabolism [3]. These factors collectively contribute to overall health and can significantly impact the development of metabolic disorders and chronic diseases.

The management of obesity involves implementing strategies, including dietary control and the use of metabolic, hormonal, or appetite-controlling medications [4]. However, weight loss achieved through dieting and the use of appetite-reducing medications often causes weight regain in the long term [5]. To address this challenge, direct targeting of adipose tissue function and the cells within the tissue has been proposed as potential effective therapeutic approaches [6,7]. Previous studies on preadipocytes of humans with obesity revealed that preadipocytes can undergo epigenetic reprogramming that alters their insulin sensitivity and adipokine signaling pathways [8]. Therefore, targeting the cellular processes within adipose tissue could have significant implications for obesity treatment and management.

*Cissus quadrangularis* L. (CQ) is a medicinal plant of the *Vitaceae* family and is native to tropical regions such as Thailand, India, Sri Lanka, Malaya, and West Africa [9]. CQ has been deemed safe for use because of its low cytotoxicity, with an LD50 of 3000 mg/kg in animals [10]. Pharmacological studies have demonstrated that CQ has multifaceted effects with anti-inflammatory, anticonvulsant, antimicrobial, anticancer, and antiosteoporotic activities, and can be used as treatment for other bone-related disorders [9]. In a study conducted on induced diabetic rats, the ethanolic extract of CQ exhibited antihyperglycemic activity by significantly reducing serum glucose levels and preventing a decrease in body weight [11]. Additionally, the hot water extract of CQ has been used as a food supplement for weight control, with a recommended dose of 300 mg/day (CQR-300) [12].

Recent research indicated that CQR-300 can inhibit lipid accumulation in 3T3-L1 adipocytes by downregulating the expression of adipogenesis proteins, including peroxisome proliferator-activated receptor γ (PPARγ), CCAAT/enhancer-binding protein α (C/EBPα), sterol regulatory element binding protein-1c (SREBP-1c), and FAS [13]. Furthermore, the antiobesity effects of CQR-300 have been observed in high-fat diet-induced obese mice to reduce body fat, decrease adipocyte size, and lower expression levels of PPARγ, C/EBPα, and SREBP-1c [14]. In a study involving human subjects, CQ treatment produced a decrease in obesity as evidenced by reductions in the circumference of waist and hip, increases in serum levels of leptin, and induction of white adipocyte browning with enhanced expression of uncoupling protein 1 (*Ucp1*) [15].

CQ contains a rich variety of phytochemical constituents, including alkaloids, proteins, vitamin C, amino acids, tannins, saponins, phenolic compounds, steroids, sterols, and terpenoids [16,17]. Phytochemical analysis of the hexane (H) extract of CQ leaves revealed the presence of lipid constituents such as eicosyl eicosanoate, tetratriacontanol, tetratriacontanoic acid, α-amyrin, and β-sitosterol [18]. Additionally, five dammarane-type triterpenes with osteogenic induction bioactivity have been isolated and identified from the H fraction of CQ through alkaline phosphatase activity guided isolation [19]. Furthermore, *n*-butanol fractions of CQ were reported to possess antiadipogenic activity to reduce adipogenesis in pre-adipocytes. This effect has been attributed to the presence of approximately 13 reported antiadipogenic compounds, along with more than 10 predicted bioactive compounds [20].

This study sought to investigate the antiadipogenic effects of fractions derived from the CQ hexane extract in mouse pre-adipocyte 3T3-L1 cells. A total of 30 fractions were tested, and among them, the lupenone-rich fraction emerged as a potent inhibitor of adipogenesis. Subsequent analysis of purified lupenone and the lupenone-rich fraction confirmed their antiadipogenic properties. The study also included gene expression analysis, along with assessments of mitochondrial density and glucose uptake.

## 2. Materials and Methods

### 2.1. Plant Materials

Fresh aerial parts of CQ aged one-and-a-half years were dried at a temperature not exceeding 60 °C. Dried stems were powdered by destruction. Powdered CQ was also kindly provided by the Chao Phraya Abhaibhubejhr Hospital, Prachin Buri, Thailand.

### 2.2. Preparation of CQ Hexane Extract by Fractionation

**Figure 1:**
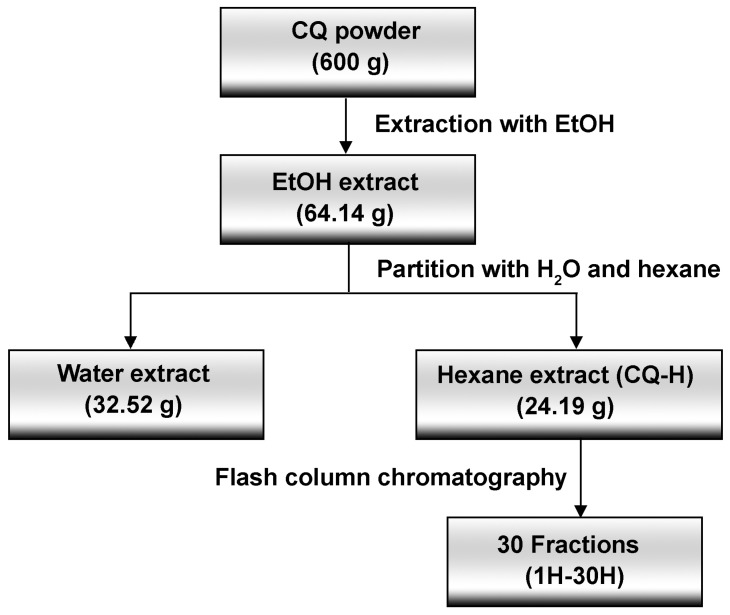
Extraction schematic of *Cissus quadrangularis* L.

A total of 600 g of CQ powder was soaked in 2.4 L of 95% ethanol (EtOH) for 3 days, then filtered to remove excess particles. Following completed extraction, the solvent was removed under reduced pressure. The extraction was repeated two more times. The crude ethanol extract was obtained as a black oil (64.14 g). The crude extract was suspended in water (200 mL) and extracted with H (3 × 400 mL). The resulting organic phase was combined and concentrated under reduced pressure to obtain the corresponding crude hexane extract (CQ-H) (24.19 g, 4.1%) as a black oil. CQ-H was fractionated in the next step. The aqueous phase was concentrated under reduced pressure to obtain a water extract as a black oil (32.52 g, 5.4%). The process of extraction and fractionation of CQ-H is shown in Figure 1.

### 2.3. Flash Column Chromatography 

The H extract (24.19 g) was coated onto silica gel and transferred to a packed silica gel column (Silica gel 60, Merck (Boston, MA, USA), 0.015–0.040 mm). The extract was fractionated by sequential elution of the column with the increasing polarity of solvents including hexane, ethyl acetate (EtOAc) and methanol to obtain 87 fractions (listed in Table 1), which were then analyzed via thin-layer chromatography (TLC). Similar fractions were combined and evaporated under reduced pressure; a total of 30 fractions, 1H–30H, were obtained. 

### 2.4. Purification of Lupenone from 2H

The 2H fraction (300 mg) was coated onto silica gel and loaded on a packed silica gel column (Silica gel 60, Merck, 0.015–0.040 mm). Subsequently, the column was eluted using a mobile phase of 1% and 2% EtOAc:hexane, respectively. Lupenone from CQ (Lu-CQ) was obtained as an off-white oil (234.2 mg) and identified by comparison analysis using TLC and nuclear magnetic resonance spectroscopy (NMR) with lupenone in agreement with the reported literature [21].

### 2.5. Cell Culture 

3T3-L1 preadipocytes were obtained from the Japanese collection of research bioresources cell bank (JCRB) (Osaka, Japan; JCRB9014, Lot#: 06182020). Cells were cultured in complete growth media (CGM) containing Dulbecco’s Modified Eagle Medium (Hyclone, Logan, UT, USA; Cat#: SH30021.02) supplemented with 10% fetal bovine serum (Hyclone, USA; Cat#: SV30160.03), 100 units/mL penicillin streptomycin (Hyclone, USA; Cat#: SV30010), and 2 mM L-glutamine (Corning Inc., Corning, NY, USA; Cat#: SH30034.02) at 37 °C in a humidified condition containing 5% CO_2_. The medium was changed every 2 days, and cells were sub-cultured to 80% confluency.

### 2.6. Adipogenic Differentiation

To induce the differentiation of 3T3-L1 preadipocytes into mature adipocytes, 3T3-L1 cells were seeded in CGM at 5 × 10^3^ cells/well in a 96-well plate for 2 days. Then, the medium was changed to adipogenic differentiation media recipe I (ADMI) containing CGM supplemented with 1 µM dexamethasone (Sigma, St. Louis, MO, USA; Cat#: D8893-1MG), 10 µg/mL insulin (Sigma, USA; Cat#: I9278-5ML), and 500 µM 3-isobutyl-1-methyxanthine (Sigma, USA; Cat#: I5879-1G). After 2 days in ADMI, the media were changed to adipogenic differentiation media recipe II (ADMII) containing CGM-supplemented 10 µg/mL insulin and cells were cultured for another 6 days [22]. Fractions of CQ-H (0.5–1.0 mg) were dissolved in 100% dimethyl sulfoxide (DMSO) (Biochemical, Fort Detrick, MD, USA; Cat#: PC0906) to prepare a stock solution at 100 mg/mL. Then, the extracts were diluted to the desired concentrations in either ADMI or ADMII. DMSO, including the vehicle control group without CQ extract, was maintained at a final concentration of 0.1% (*v*/*v*).

### 2.7. Oil Red O Staining

Oil red O staining was used to detect intracellular lipid droplet accumulation. Briefly, 3T3-L1 cells were fixed with 4% paraformaldehyde at room temperature for 10 min. Subsequently, cells were washed twice with phosphate-buffered saline (PBS) and incubated with 60% isopropanol for 5 min. Cells were then stained with a 0.5% (*w*/*v*) oil red O solution in 60% isopropanol (Sigma, USA; Cat#: O1391) for 10 min at 37 °C. Cells were then extensively washed with PBS to remove excess dye [23]. Microscopic images were obtained using an inverted microscope (Leica, DMi1, Wetzlar, Germany) from five different areas for each replicate. The area of the stained lipid droplets was quantified using ImageJ (National Institutes of Health, Bethesda, MD, USA) [24]. 

### 2.8. Cell Viability Assay

The alamarBlue cell viability assay was used to determine cell viability. The 3T3-L1 cells were seeded at 5 × 10^3^ cells per well in 96-well plates. At the end of the experiment, the medium was replaced with 120 μL of CGM containing 10% alamarBlue reagent (Invitrogen, Waltham, MA, USA; cat#: DAL1025) and incubated for 3 h at 37 °C. The absorbances at 570 and 600 nm were determined using a microplate reader (PACKARD, A153601, Palo Alto, CA, USA). Absorbance values for 570 and 600 nm were used for percentage reduction calculation as follows [25] and the percentage of cell viability was calculated as a percentage compared with the vehicle-treated control [26].

$$\%\;Reduction=\frac{\left(E_{ox600}\times A_{570}\right)-\left(E_{ox570}\times A_{600}\right)\times 100}{\left(E_{red570}\times A_{600\;t0}\right)-\left(E_{red600}\times A_{570\;t0}\right)}$$

E_ox570_ = E of oxidized alamarBlue Reagent at 570 nm = 80,586
E_ox600_ = E of oxidized alamarBlue Reagent at 600 nm = 117,216
E_red570_ = E of reduced alamarBlue Reagent at 570 nm = 155,677
E_red600_ = E of reduced alamarBlue Reagent at 600 nm = 14,652
A_570_ = absorbance of test wells at 570 nm
A_600_ = absorbance of test wells at 600 nm
A_570 t0_ = absorbance of negative control well at 570 nm
A_600 t0_ = absorbance of negative control well at 600 nm

### 2.9. Glycerol-3-Phosphate Dehydrogenase (GPDH) Activity Assay

GPDH, a cytoplasmic enzyme, plays a crucial role in the biosynthesis of triglycerides. It catalyzes the conversion of dihydroxyacetone phosphate into glycerol 3-phosphate. Cells were lysed with buffer containing 20 mM Tris (Amresco, Boise, ID, USA; cat#: 1185-53-1), 1 mM EDTA (Fluka, Buchs, Switzerland; cat#: 34549), and 1 mM β-mercaptoethanol (ITW Reagents, Hessen, Germany; cat#: 60-24-2) for 3 min, scraped with pipette tips, and transferred to fresh microcentrifuge tube, and sonicated for 5 min. The cell debris was centrifuged, and the supernatants were aliquoted and stored at −20 °C. Samples were thawed at room temperature, and 100 μL per well added to 96-well plates. Exactly 100 μL of the enzyme reaction buffer, containing 0.1 M thiethanolamine (CARLO ERBA, Cornaredo, Italy; cat#: 102-71-6), 2.5 mM EDTA, 0.1 mM β-mercaptoethanol, and 90 μL 334 µM NADH (Sigma, USA; cat#: N8129), was added and incubated for 10 min at 37 °C before adding 10 μL 4 mM DHAP (Sigma, USA; cat#: 51269). The absorbance was measured at 340 nm every 5 min for 30 min (ΔOD340 nm/min) [27]. The GPDH activity was calculated using the absorbance value and reaction duration time with the following equation:

$$GPDH=\frac{(∆A340/min\times 100\;µL)\times sample\;dilution}{\left(6.22\;\times \;10\;µL\right)}$$

### 2.10. BODIPY and DAPI Staining

Fluorinated boron-dipyrromethene (BODIPY) and 4’,6-diamidino-2-phenylindole (DAPI) staining techniques were used to analyze the size of lipid droplets and the number of nuclei, respectively. Briefly, cells were fixed with 4% paraformaldehyde for 5 min and permeabilized with 0.1% Triton X-100 for 10 min. Cells were then stained with 10 µg/mL of BODIPY (Sigma, USA; Cat#: 121207) for 15 min, followed by incubation with 1 µg/mL of DAPI (Sigma, USA; Cat#: 28718) for 5 min. Images were captured using an inverted fluorescence microscope (Olympus, CK30, Tokyo, Japan) from five different areas per replicate. The number of nuclei and the size distribution of lipid droplets were analyzed using CellProfiler (Broad Institute, Cambridge, MA, USA). Lipid droplet size was determined by calculating the average of the minimum and maximum Feret diameter [28]. 

### 2.11. Mitochondrial Staining

For mitochondrial staining, 3T3-L1 cells were incubated with 200 nM MitoNIR (Abcam, Waltham, MA, USA; cat#: AB176833) in PBS solution for 1 h at 37 °C. Cells were then washed twice with PBS and counterstained with 0.1 μg/mL of Hoechst (Abcam, Waltham, MA, USA; cat#: 228551) for 5 min. Fluorescence imaging was conducted using a fixed exposure time of 100 ms for the blue channel (Hoechst dye) and 400 ms for the green channel (MitoNIR). The mitochondrial density was analyzed by calculating the mean of the corrected total cell fluorescence (CTCF) within the entire cell area of the image, and the CTCF was analyzed per individual cell. A total of at least 200 cells per group were analyzed. The categorization of cells was based on their lipid droplet phenotypes. The non-differentiated (ND) group consisted of cells without visible lipid droplets. Cells with less than 10% of the cell area covered by lipid droplets were classified as having low levels of differentiation (LD). The big droplets (BD) group included cells with at least one lipid droplet larger than 2.2 μm in diameter. Cells without large lipid droplets were assigned to the small droplets (SD) group. The fluorescence intensity was measured using ImageJ as previously described [29]. Firstly, the area of interest was selected, and subsequently, the measure tool in the analyze menu of ImageJ was employed to obtain measurements of the integrated density and mean grey value for both the fluorescence signal and the corresponding background area. These measurements were utilized in the calculation of CTCF using the following equation:

$$Mean\;of\;CTCF=Integrated\;density-\left(area\;of\;selected\;cell\times mean\;fluorescence\;of\;background\right)$$

### 2.12. Gene Expression Analysis

3T3-L1 cells were seeded at 2 × 10^4^ cells/well in a 24-well plate and cultured with the conditioned media containing CQ-H at a concentration of 80 µg/mL for 8 days. Total ribonucleic acid (RNA) was extracted using the total RNA mini kit (Favorgen, Ping Tung, Taiwan; cat#: FATRK001). Complementary DNA was synthesized using the iScript reverse transcription supermix (Biorad, Hercules, CA, USA; cat#: 1708841) following the manufacturer’s protocols. Quantitative polymerase chain reaction (PCR) was performed with the iTaq Universal SYBR Green Supermix (Biorad, Hercules, CA, USA; cat#: 1725122) using the Applied BiosystemsTM 7500 real-time PCR system. Melting curve analysis was performed to confirm the amplification of the target gene. The gene names and corresponding primer sequences are listed in Table 2. Gene expression levels were determined using the 2^−ΔΔCT^ method with 18s normalization, and a cut-off of two-fold change was applied [30]. 

### 2.13. Glucose Consumption 

Glucose levels in the culture media were analyzed at four time points, after 24 and 48 h, in ADMI (stage I) and ADMII media (stage II). Media were supplemented with either 2H or Lu-CQ at 80 µg/mL. Glucose concentrations were measured with an Accu-Chek blood glucometer and test strip (Roche, Karlsruhe, Mannheim, Germany) [31]. To adjust the glucose concentration within the reading value of the glucometer (0.6–33.3 mmol/L), cultured media were mixed with basal DMEM and distilled water in a 1:1:1 ratio. The glucometer reading value was calibrated using a known glucose content from a generated standard curve (Figure S1). The percentage of glucose content representing the remaining glucose after consumption at the collected time point was calculated by comparing it with the initial fresh media.

### 2.14. Statistical Analysis

Data are presented as the mean ± standard error (SE) from triplicates. SPSS V25 statistical software (IBM Corp., Armonk, NY, USA) was used to analyze the data in this study. All statistical differences between the mean values of each group were analyzed using one-way analysis of variance (ANOVA) with the least significant difference (LSD) multiple-comparison test and Duncan’s test. *p* < 0.05 was considered significant.

## 3. Results

### 3.1. Fractionation of CQ-H

Flash column chromatography, in combination with TLC, was used to separate the CQ-H extracts as described in Section 2.3. The composition of each fraction was analyzed by TLC. The identical components were then combined and evaporated under low pressure at 45 °C. The numbers were ordered in sequence from the first to the last extraction based on their increasing polarity. The percentage yields of the 30 fractions (1H–30H) are shown in Table 3.

### 3.2. Antiadipogenesis of CQ-H Fractions

**Figure 2:**
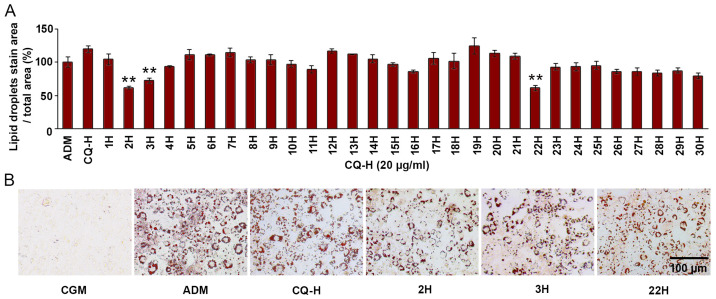
Three of the thirty CQ hexane fractions (20 µg/mL) decreased accumulation of lipid droplets in 3T3-L1 cells. (**A**) Quantitative result of lipid droplet staining shown as a percentage of lipid droplet area. (**B**) Microscopic images depicting undifferentiated 3T3-L1 cells cultured in complete growth media (CGM), differentiated cells in adipogenic differentiation media (ADM), and the reduction in intracellular lipid droplets observed in 3T3-L1 cells treated with fractions 2H, 3H, and 22H in ADM. Values are presented as mean ± standard error. ** Statistically significant (*p* < 0.01).

The adipogenesis inhibitory effect of the 30 CQ-H fractions (at 20 µg/mL) was assessed using 3T3L-1 cells. Differentiation of 3T3-L1 cells by ADM, compared to undifferentiated cells in CGM, was confirmed through oil red O staining and the upregulation of adipogenic markers (Figure S2 and Table S1). Consequently, fractions 2H, 3H, and 22H exhibited significant inhibition of lipid droplet formation, with reductions of 38.33 ± 2.31%, 27.58 ± 4.08%, and 38.56 ± 3.96%, respectively (Figure 2A). The reduction in lipid formation was demonstrated by a decrease in the area stained with oil red O, a decrease in the lipid droplet size, and an increase in the undifferentiated cell number (Figure 2B). 

### 3.3. Lupenone-Rich Fractions Reduced Adipogenesis

**Figure 3:**
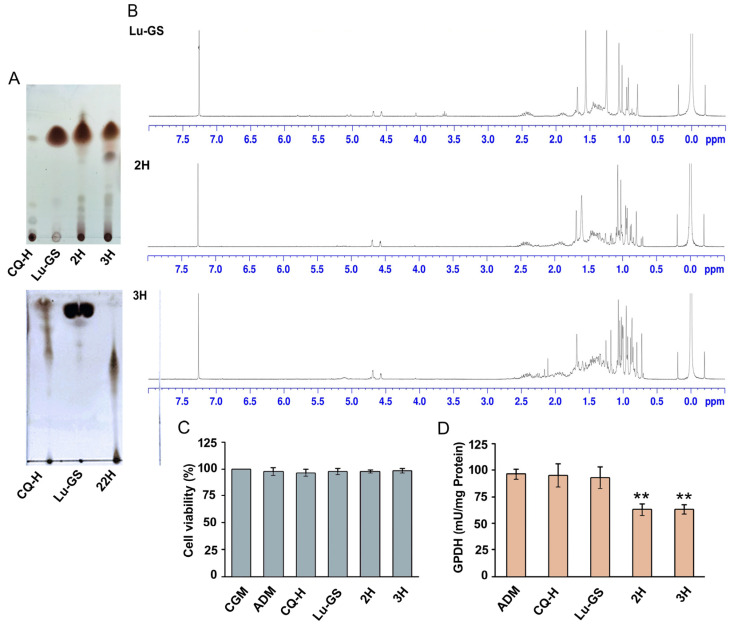
Identification of lupenone as the main component in 2H and 3H fractions. (**A**) TLC profile. (**B**) ^1^H NMR spectroscopic data of compounds in comparison with those of lupenone from GS (Lu-GS). (**C**) 3T3-L1 cell viability was determined using the alamarBlueTM assay on day 8. (**D**) GPDH activity was analyzed on day 8. Data are presented as mean ± standard error of at least three independent experiments in (**C**,**D**). ** Statistically significant (*p* < 0.01).

TLC analysis showed that 22H contained trace amounts of various compounds, whereas 2H and 3H were predominantly composed of lupenone (Figure 3A). To confirm the presence of bioactive constituents in these fractions, the 2H and 3H fractions were analyzed using NMR, and the results were compared with those of lupenone from *Gardenia saxatilis* (Lu-GS) [32]. The NMR results confirmed lupenone as the main constituent in both fractions (Figure 3B). 

Fractions 2H and 3H were selected for further investigation because they had a strong adipogenesis inhibitory effect and a high percentage yield and contained lupenone as the main constituent. The cytotoxicity and adipogenesis inhibitory effects of these fractions, as well as CQ-H and Lu-GS, were evaluated at 20 µg/mL. No cytotoxicity was observed (Figure 3C). Moreover, fractions 2H and 3H produced substantial reductions in GPDH enzymatic activity, with decreases of 34.89 ± 4.38% and 34.55 ± 6.82%, respectively (Figure 3D). The decrease in GPDH activity suggests a potential disruption in the adipogenic process, which could affect the accumulation of triglycerides and the development of mature adipocytes. These results indicate that the components present in fractions 2H and 3H may have a negative impact on triglyceride biosynthesis and adipocyte maturation. In contrast, neither the CQ-H extract nor lupenone from GS displayed any discernible inhibitory effect on adipogenesis (Figure 3D).

**Figure 4:**
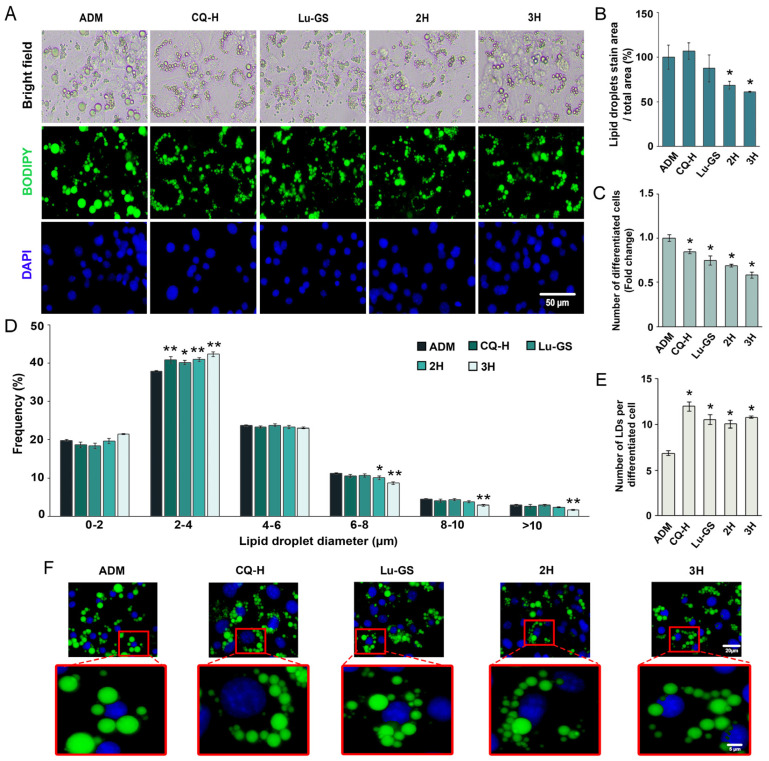
Detection of differentiated 3T3-L1 adipocytes. (**A**) Microscopic images of 3T3-L1 showing cell morphology (bright field), lipid droplet distribution (BODIPY), and nuclei (DAPI). (**B**) Percentage of lipid droplet-stained area. (**C**) Ratio of differentiated cells to the total cell number. (**D**) Distribution of lipid droplet size. (**E**) Number of accumulated lipid droplets per cell. (**F**) Characteristics of intracellular lipids showing differences in lipid droplet size. Values are presented as mean ± standard error. Statistically significant * *p* < 0.05, ** *p* < 0.01 compared to ADM control.

Fractions 2H and 3H exhibited a significant inhibitory effect on adipogenesis, producing a noticeable reduction in both the number of differentiated cells and size of lipid droplets (Figure 4A). This observation was supported by the quantitative analysis of BODIPY/DAPI staining, which allowed for the identification of cell numbers and differentiation status. The BODIPY/DAPI staining results were consistent with the observations from the oil red O staining, confirming the lower accumulation of lipid droplets in cells treated with fractions 2H and 3H than that in cells in the ADM control group (Figure 4B). By contrast, neither CQ-H nor Lu-GS produced any noticeable reduction in the lipid-stained area (Figure 4B). However, when lipid droplet formation was analyzed in conjunction with nuclei, treatment with CQ-H, Lu-GS, 2H, and 3H was observed to significantly decrease the number of differentiated cells (Figure 4C). Distribution analysis of lipid droplet size revealed that the CQ-H, Lu-GS, 2H, and 3H significantly increased the ratio of small-sized lipid droplets within the range of 2–4 μm. Additionally, 2H treatment significantly decreased the ratio of large lipid droplets within the range of 6–8 μm, and 3H decreased the size of lipid droplets >6 μm (Figure 4D). The increase in the number of lipid droplets per cell agreed with the increase in the number of small lipid droplets induced by CQ-H, Lu-GS, 2H, and 3H (Figure 4E,F).

### 3.4. Lupenone and Lupenone-Rich CQ-H Fraction Increased Mitochondrial Density in 3T3-L1 Cells 

**Figure 5:**
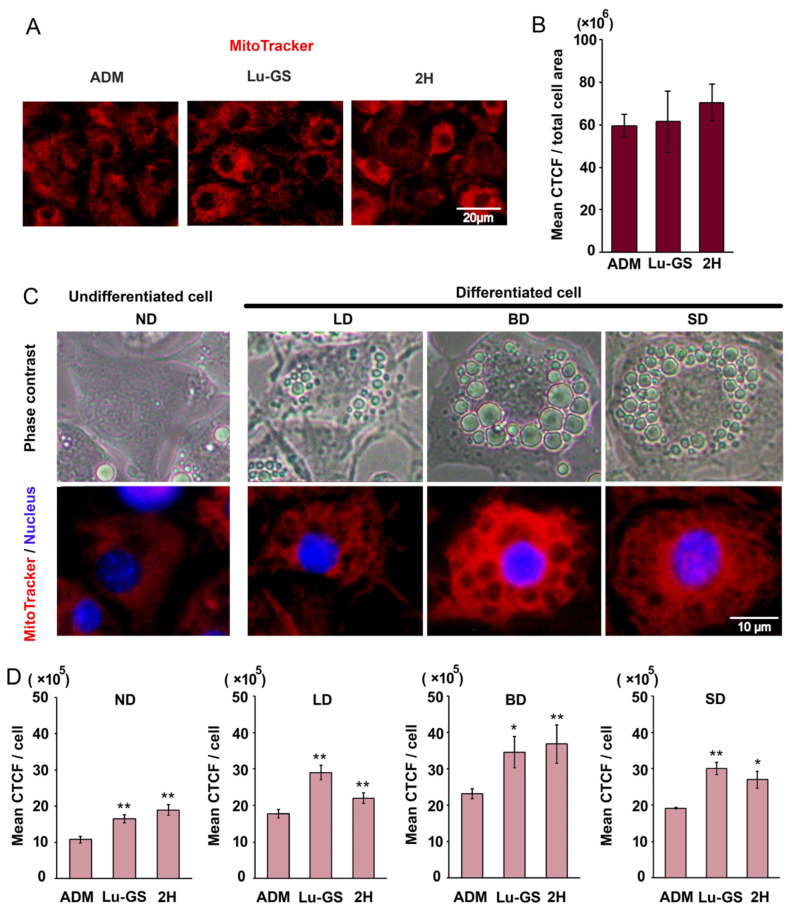
Cellular effects of CQ on mitochondrial density in 3T3-L1 cells. Cells were treated with lupenone from *Gardenia saxatilis* (Lu-GS) and fraction 2H from CQ hexane extract concentration at 20 µg/mL. (**A**) Mitotracker staining showed mitochondrial distribution in red fluorescence. (**B**) Mean CTCF values. (**C**) Differences in mitochondrial density of cells categorized by the stage of adipogenic differentiation: ND = non-differentiated, LD = low levels of differentiation, BD = differentiated cells with big lipid droplets, SD = differentiated cells with small lipid droplets. (**D**) Mean CTCF values of the mitochondrial density for each cell type. Values are presented as mean ± standard error. Statistically significant * *p* < 0.05, ** *p* < 0.01 compared to ADM control.

Treatment with lupenone and the lupenone-rich fraction 2H was hypothesized to be involved in brown adipogenesis and triggered cell biogenesis because of the observed increase in small lipid droplets. To explore and validate this hypothesis, the mitochondrial density was monitored using CytoPainter MitoNIR. The results showed that treatment with Lu-GS and 2H increased the mitochondrial density in several cells as indicated by the bright red cells shown in Figure 5A. However, when fluorescence intensity was analyzed using CTCF, a high variation was observed in the Lu-GS and 2H groups, and no statistical difference was present in comparison with the control group (Figure 5A,B).

Further investigation revealed that the high variation in mitochondrial density within the treatment group was caused by differences in the degree of adipogenic differentiation of the cells. ND cells and LD cells had minimal mitochondrial density, whereas highly differentiated cells contained both BD and SD and had high mitochondrial density (Figure 5C). The CTCF was reanalyzed and categorized based on the degree of adipogenic differentiation and showed that Lu-GS and 2H increased the mitochondrial density in each categorized cell (Figure 5D).

### 3.5. Extraction and Identification of Lupenone from 2H Fraction

**Figure 6:**
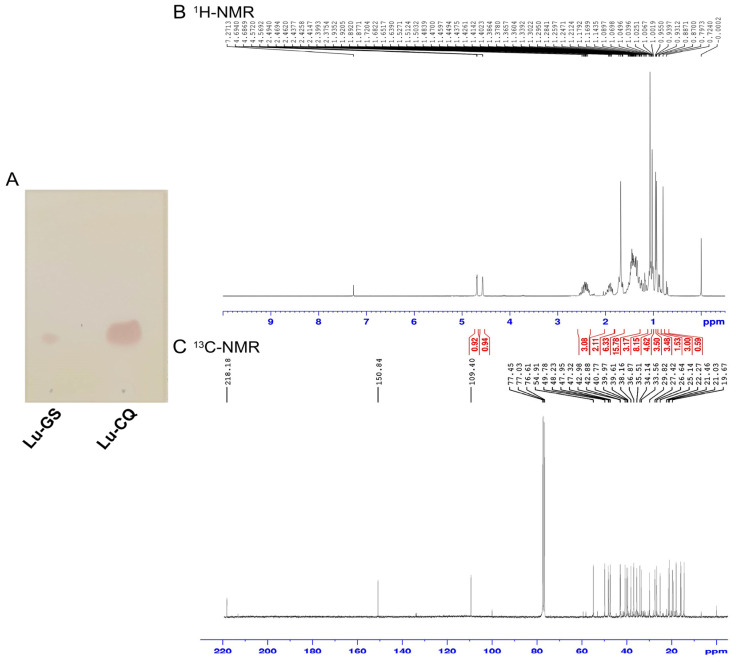
Identification of Lu-CQ. (**A**) Chromatographic profile by TLC. (**B**) ^1^H NMR spectroscopic data of Lu-CQ. (**C**) ^13^C NMR spectroscopic data of Lu-CQ.

The TLC results and anti-adipogenesis effect of 2H and lupenone led to a hypothesis that 2H might have been primarily composed of lupenone. Purification of 2H was performed to confirm this hypothesis. Lupenone (234.2 mg) was extracted from 2H (300 mg) and its identity was confirmed. TLC was performed to compare lupenone extracted from 2H (Lu-CQ) with Lu-GS (Figure 6A). To characterize Lu-CQ, the ^1^H and ^13^C NMR spectra were compared with literature data [21]. The ^1^H NMR spectrum of Lu-CQ exhibited peaks of olefinic protons (H-29) (Figure 6B), and the ^13^C NMR spectrum showed peaks at 218.2, 150.8, and 109.4, confirming carbonyl carbon (C-3), olefinic carbon (C-20 and C-29), respectively (Figure 6C). Carbon multiplicity was then confirmed via ^1^H and ^13^C-NMR, and the positions of carbon atom were related to protons. The structure was elucidated by comparing the spectral data with reference standards [21] (Table 4).

**Figure 7:**
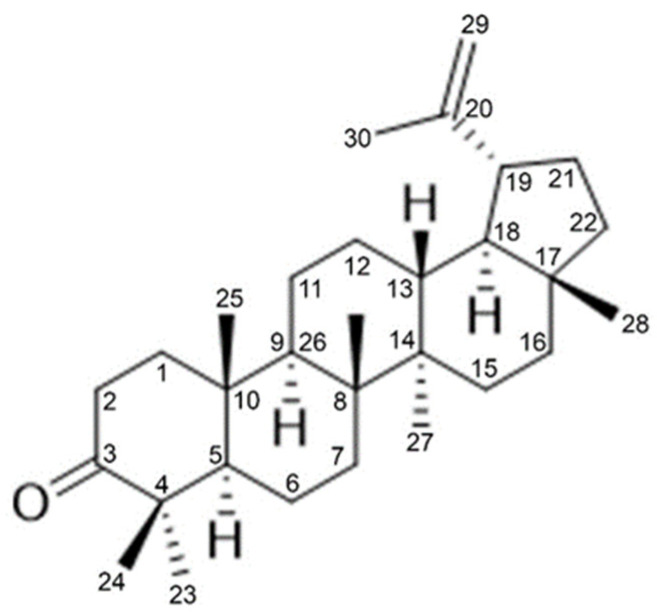
Structure of Lu-CQ based on NMR results.

Lupenone was also identified by comparing the spectral data with the literature. The structure of Lu-CQ was found to be similar to the reference standards of lupenone in agreement with the reported data [21] (Figure 7).

### 3.6. Higher Adipogenic Inhibitory Effect of the 2H Fraction Compared with Lu-CQ

**Figure 8:**
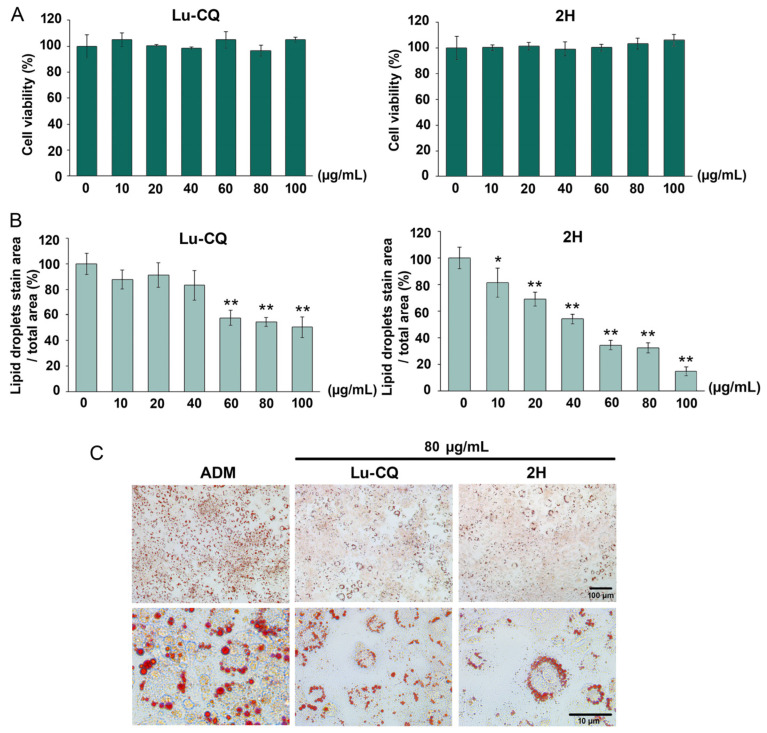
Comparative analysis of antiadipogenic effects of 2H and Lu-CQ. (**A**) Cell viability. (**B**) Quantitative result of lipid droplet staining shown as a percentage of lipid droplet area. (**C**) Microscopic images showing reduction in intracellular lipid droplets in 3T3-L1 cells treated with Lu-CQ and 2H. Values are presented as mean ± standard error. Statistically significant * *p* < 0.05, ** *p* < 0.01 compared to ADM control.

Following the extraction of Lu-CQ from the 2H fraction, a comparative evaluation was conducted to assess the respective inhibitory effects on adipogenesis between 10 and 100 µg/mL. Both Lu-CQ and 2H exhibited a dose-dependent inhibition of 3T3-L1 adipogenesis while simultaneously preserving cell viability (Figure 8A). Notably, Lu-CQ exhibited a significant antiadipogenic effect between 60 and 100 µg/mL, producing inhibition rates ranging from 42.20 ± 5.95% to 49.60 ± 8.20% (Figure 8B). Conversely, the 2H fraction displayed higher potency, exhibiting inhibitory effects at concentrations between 10 and 100 µg/mL and yielding inhibition rates spanning from 30.87 ± 5.40% to 85.21 ± 3.48% (Figure 8B).

### 3.7. Downregulation of Expression of Adipogenic Master Gene with Increased Expression of Glucose Transporter Genes Induced by Lu-CQ and 2H

**Figure 9:**
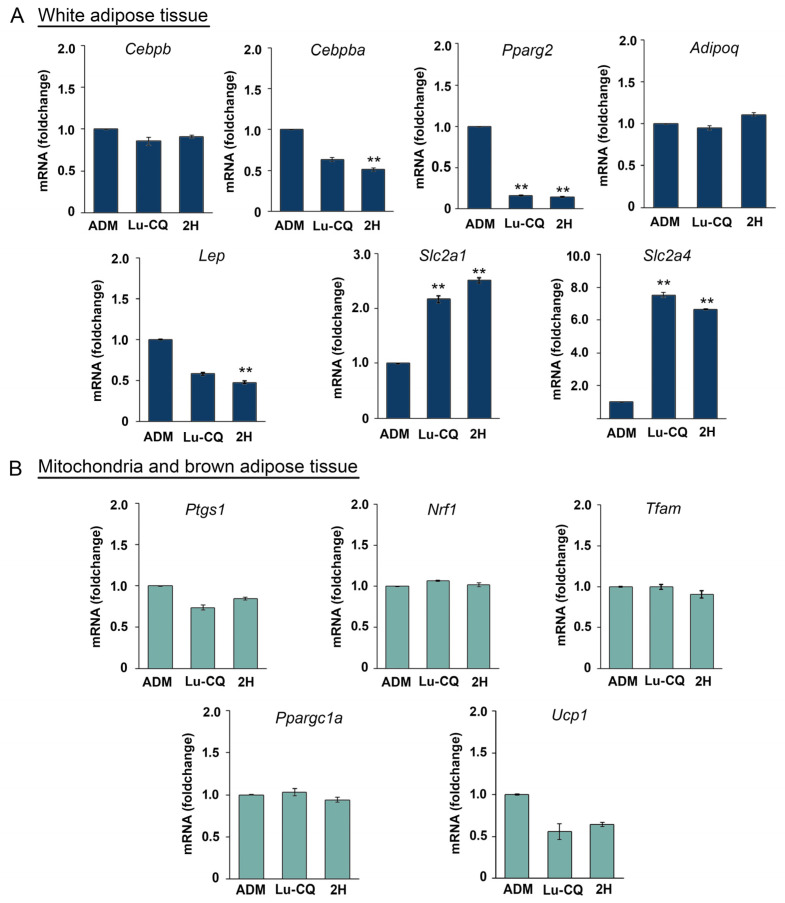
Relative gene expression of adipogenesis-related genes in 3T3-L1 cells. Cells were treated with fraction 2H from CQ hexane extract and lupenone isolated from 2H (Lu-CQ) at 80 µg/mL. (**A**) Expression of genes associated with white adipose tissue formation and function. (**B**) Expression of genes associated with mitochondrial function and brown adipose tissue function. Values are presented as the mean ± standard error. Significant differences are indicated as ** *p* < 0.01, using a 2-fold cut-off.

Treatment of 3T3-L1 cells with Lu-CQ and 2H significantly influenced gene expression associated with adipogenesis. Specifically, the expression of peroxisome proliferator-activated receptor gamma 2 (*Pparg2*), a key regulator of adipogenesis, was downregulated by 6.10- and 6.71-fold with Lu-CQ and 2H treatment, respectively (Figure 9A). Furthermore, treatment with 2H significantly decreased the expression of *Cebpa* and leptin (*Lep*) (Figure 9A). However, no significant changes were observed in the expression of adiponectin (*Adipoq*) and CCAAT/enhancer-binding protein beta (*Cebpb*) (Figure 9A). Interestingly, both Lu-CQ and 2H treatment upregulated the expression of glucose transporter genes, specifically solute carrier family 2 (facilitated glucose transporter), member 1 (*Slc2a1*) and member 4 (*Slc2a4*) (Figure 9A). Lu-CQ treatment increased the expression of *Slc2a1* by 2.17-fold while 2H increased this by 2.52-fold. Similarly, Lu-CQ treatment increased the expression of *Slc2a4* by 7.53-fold, while 2H treatment increased the expression 6.65-fold (Figure 9A). These findings suggest that both Lu-CQ and 2H may enhance glucose uptake in 3T3-L1 cells by upregulating the expression of glucose transporter genes *Slc2a1* and *Slc2a4*.

We then investigated the effect of Lu-CQ and 2H on the expression of mitochondrial-related genes and uncoupling protein 1 gene (*Ucp1*) on the basis of our findings and a previous study on CQ in human white adipocytes [15]. However, the expression of prostaglandin-endoperoxide synthase 1 (*Ptgs1*), nuclear respiratory factor 1 (*Nrf1*), transcription factor A mitochondrial (*Tfam*), *Ucp1* and peroxisome proliferative activated receptor gamma coactivator 1 alpha gene (*Ppargc1a*) was not significantly affected (Figure 9B). 

The average mitochondrial density remained unchanged, as confirmed by the increased extract concentrations at 80 µg/mL, and the presence of similar high variations among cells at each differentiated stage (Figure 5B and Figure S3A,B). Notably, a higher mitochondrial density was observed in highly differentiated cells with big lipid droplets (BD) and small lipid droplets (SD). However, when we analyzed each subpopulation, we found that treatment with Lu-CQ and 2H resulted in an increase in mitochondrial density in these subpopulations, while reducing the number of BD and SD cells (Figure S3C). Although there was an increase in mitochondrial density within each specific subpopulation due to the Lu-CQ and 2H treatment, this increase was offset by the higher number of non-differentiated (ND) and low-differentiated (LD) cells in the overall cell population. As a result, the average mitochondrial density across the entire cell population remained unchanged. These findings were consistent with the qPCR results.

### 3.8. Lu-CQ and 2H Increased Glucose Consumption during Adipogenesis of 3T3-L1

**Figure 10:**
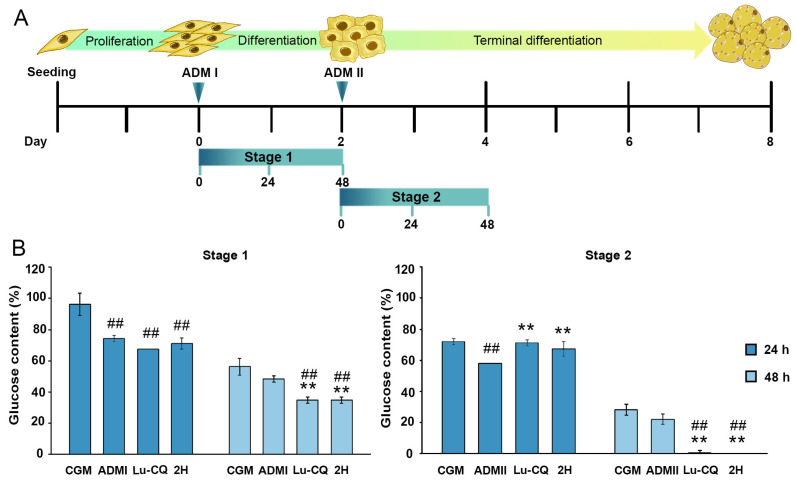
Decreased media glucose content by fraction 2H from CQ hexane extract and lupenone isolated from 2H (Lu-CQ) at 80 µg/mL during 3T3-L1 differentiation. (**A**) Schematic diagram illustrating the stages of differentiation, media implementation, and time points for glucose measurements. Glucose content was analyzed at stage 1 and stage 2 of adipogenic differentiation after 24 and 48 h of induction with ADMI and ADMII, respectively. (**B**) Percentage of glucose content in media compared the fresh media at 0 h. Values are presented as mean ± standard error. Significant differences are indicated as ^##^ *p* < 0.01 compared with CGM, and ** *p* < 0.01 compared with ADMI or ADMII.

As Lu-CQ and 2H influenced the expression of two glucose transporter genes, the glucose consumption was further analyzed. This analysis was conducted in two stages: at 24 and 48 h after changing the media to ADMI and ADMII (Figure 10A). Growth in ADMI significantly increased the glucose consumption of 3T3-L1 cells during the initial 24 h with more than a 22% drop in glucose content in the cultured media (Figure 10B). The addition of Lu-CQ and 2H did not further enhance glucose consumption in ADMI during this period (Figure 10B). However, after 48 h in the first stage, Lu-CQ and 2H significantly increased glucose consumption and reduced media glucose content by more than 10% (Figure 10B). The second stage of differentiation also yielded similar outcomes, with Lu-CQ and 2H enhancing glucose consumption after 48 h in ADMII by around 22% (Figure 10B).

## 4. Discussion

The use of CQ in the management and treatment of obesity and metabolic syndrome has shown promising outcomes in various human studies [33]. Notably, the administration of CQR-300 has directly resulted in a significant reduction in body fat (up to 12.8%) after an 8-week intervention [12]. Moreover, significant improvements in blood parameters associated with metabolic syndrome were observed following an 8-week treatment regimen with CQR-300. These improvements include a decrease in blood pressure, triglyceride levels, and fasting blood glucose, accompanied by an increase in serotonin levels [34]. CQ treatment of obese individuals for 8 weeks in a recent study produced significant reductions in waist and hip circumference while also increasing serum leptin levels [15]. The study further suggested that CQ treatment could enhance white adipocyte browning, as evidenced by the upregulation of *Ucp1* mRNA expression, and a simultaneous decrease in glycerol release [15]. These findings underscore the potential of CQ as a valuable therapeutic approach for managing obesity and balancing metabolic activity. However, the active components and underlying mechanisms responsible for its therapeutic effects remain relatively unknown. Therefore, we conducted a comprehensive analysis on 30 distinct fractions isolated from CQ hexane extracts to explore their potential antiadipogenic effects on mouse preadipocytes. 

Among the 30 CQ-H fractions analyzed, fractions 2H and 3H exhibited marked antiadipogenic activity and were consequently selected for further analysis. TLC and NMR analyses conclusively identified lupenone as a major constituent in both fractions. Although lupenone has been recognized as a bioactive component in CQ [35], its role as an antiadipogenic compound in CQ has not yet been established. Lupenone from rhizome of *Musa basjoo* displayed antidiabetic effects by reducing fasting blood glucose and glycated hemoglobin levels in diabetic rats [36]. Additionally, lupenone derived from *Adenophora triphylla* had inhibitory effects on the differentiation of 3T3-L1 adipocytes. The study revealed that 100 and 200 µM concentrations of lupenone effectively downregulated the expression of PPARγ2 and resistin mRNA. However, a higher concentration of 200 µM was necessary to observe the downregulation of the expression of mRNA for the adipocyte fatty acid-binding protein C/EBPα [37].

In our study, 20 µg/mL of the fractions was used for antiadipogenic screening to find the potent bioactive compounds. The 2H and 3H fractions rich in CQ-lupenone substantially reduced the enzymatic activity of GPDH without any cytotoxic effects. Furthermore, they effectively suppressed adipogenesis in 3T3-L1 cells, which consequently increased the proportion of undifferentiated cells, decreased the lipid droplet size, and increased mitochondrial density when cells were characterized by their adipogenic differentiation. In the initial experiment, the effects of Lu-GS [32] were compared with those of 2H and 3H. Lu-GS had a similar effect on lipid droplet formation, decreasing the number of differentiated cells and lipid droplet size. However, Lu-GS had lower potency than that of 2H and 3H and did not significantly reduce overall lipid accumulation or GPDH activity. The experimental results indicate that the phytochemical constituents present in 2H and 3H, in addition to their associated components, may exhibit synergistic effects in decreasing lipid droplet accumulation and GPDH activity. These findings suggest that higher concentrations of lupenone may be required to achieve its maximum effectiveness.

After successfully isolating lupenone from 2H, additional experiments were conducted to compare its antiadipogenic effects and explore the underlying mechanisms. The potency of 2H was higher than that of Lu-CQ, with observable antiadipogenesis observed at 10 µg/mL for 2H and 60 µg/mL (141 µM) for Lu-CQ. This confirmed the remarkable effectiveness of 2H, which surpassed that of purified lupenone. While lupenone constitutes a major portion of 2H (78% yield), its combination with other compounds with similar chemical properties could enhance its potency. Such potent effectiveness can be attributed to the synergistic effects commonly observed in plant extracts [38]. Notably, numerous naturally occurring compounds with similar chemical structures to lupenone have been identified and categorized as lupane derivatives. These lupane derivatives can be classified into three main groups of lupeol, betulin, and betulinic acid derivatives [39]. In the case of lupenone, this is grouped with 12 other compounds as lupeol derivatives [39]. Previous studies have reported the antiadipogenic properties of these lupane derivatives, including lupeol and betulinic acid, highlighting their potential in this regard [40,41,42,43].

Lu-CQ and 2H strongly inhibited expression of the key activator of adipogenesis, *Pparg2*, while increasing the expression of glucose transporter genes *Slc2a1* and *Slc2a4*. This enhancement in gene expression was noted following the improved cellular glucose uptake upon treatment with Lu-CQ and 2H. Previous studies reported the beneficial effects of CQ extract and lupenone on diabetic treatment, including an amelioration in insulin resistance and a reduction in fasting blood glucose levels [36,44,45]. Additionally, synthetic PPARγ ligands, which competitively block thiazolidinedione-induced PPARγ activation and adipogenic differentiation, can increase glucose uptake in 3T3-L1 cells [46]. In addition, other PPARγ antagonists, such as betulinic, have been identified and could enhance glucose uptake while inhibiting adipogenesis [40,42,47].

## 5. Conclusions

This study demonstrated the potential of CQ as a therapeutic approach for managing obesity and promoting metabolic balance. We successfully fractionated the CQ hexane extract into 30 fractions and identified lupenone-rich fractions 2H and 3H as potent inhibitors of adipogenesis. Treatment of mouse preadipocytes with these fractions effectively reduced the number of differentiated cells and decreased the intracellular lipid droplet size. We also revealed that differentiated cells treated with 2H exhibited higher mitochondrial density, suggesting a potential role in cellular metabolism. Lu-CQ exhibited dose-dependent inhibition of adipogenesis, with 2H demonstrating greater potency. Notably, lupenone constitutes a major component in 2H, signifying its substantial contribution to the observed effects. These findings underscore the necessity for further investigations into lupenone and its potential chemical derivatives, as well as exploring the potential synergistic effects when combined with other compounds present in 2H. Moreover, Lu-CQ and 2H treatment downregulated the mRNA expression of *Pparg2*, a key regulator of adipogenesis, while upregulating that of glucose transporter genes (*Slc2a1* and *Slc2a4*), indicating an improvement in glucose uptake. Overall, these findings illuminate the role of lupenone-rich fractions in CQ that contributes to the regulation of metabolic activity and the suppression of adipose tissue formation. Further research and exploration of the therapeutic potential of lupenone and its derivatives in CQ could enable the development of novel interventions for obesity management and metabolic disorders.

## Figures and Tables

**Table 1:** Volumes of solvent (mobile phase) obtained via column elution by flash column chromatography.

No.	Solvent	Times	Volume (mL)	Fractions
1.	100% Hexane	5	2500	1–5
2.	1% Ethyl acetate/Hexane	5	2500	6 –10
3.	2% Ethyl acetate/Hexane	5	2500	11–15
4.	3% Ethyl acetate/Hexane	6	3000	16–21
5.	4% Ethyl acetate/Hexane	7	3500	22–28
6.	5% Ethyl acetate/Hexane	7	3500	29–35
7.	6% Ethyl acetate/Hexane	6	3000	36–41
8.	7% Ethyl acetate/Hexane	5	2500	42–46
9.	9% Ethyl acetate/Hexane	3	1500	47–49
10.	12% Ethyl acetate/Hexane	3	1500	50–52
11.	20% Ethyl acetate/Hexane	3	1500	53–55
12.	25% Ethyl acetate/Hexane	3	1500	56–58
13.	30% Ethyl acetate/Hexane	3	1500	59–61
14.	35% Ethyl acetate/Hexane	3	1500	62–64
15.	40% Ethyl acetate/Hexane	2	1000	65–66
16.	50% Ethyl acetate/Hexane	3	1500	67–69
17.	60% Ethyl acetate/Hexane	3	1500	70–72
18.	80% Ethyl acetate/Hexane	3	1500	73–75
19.	90% Acetate/Hexane	3	1500	76–78
20.	100% Ethyl acetate	2	1000	79–80
21.	2% Methanol/Ethyl acetate	1	500	81
22.	5% Methanol/Ethyl acetate	1	500	82
23.	10% Methanol/Ethyl acetate	3	1500	83–85
24.	100% Methanol	2	1000	86–87

**Table 2:** Gene name and the primer sequence.

Gene Symbol	Gene Name	Forward Primer (5′–3′)	Reverse Primer (5′–3′)
18S	18S ribosomal RNA	5′-ACCGCAGCTAGGAATAATGGA-3′	5′-GCCTCAGTTCCGAAAACCA-3′
*Adipoq*	Adiponectin	5′-GATGCAGGTCTTCTTGGTCCTAA-3′	5′-GGCCCTTCAGCTCCTGTC-3′
*Cebpb*	CCAAT/enhancer-binding protein beta	5′-GCGCACCGGGTTTCG-3′	5′-GCGCTCAGCCACGTTTG-3′
*Cebpa*	CCAAT/enhancer-binding protein alpha	5′-GAGCCGAGATAAAGCCAAACA-3′	5′-CGGTCATTGTCACTGGTCAACT-3′
*Lep*	Leptin	5′-TCCCTGCCTCAGACCAGTG-3′	5′-TAGAGTGAGGCTTCCAGGACG-3′
*Nrf1*	Nuclear respiratory factor 1	5′-AGCACGGAGTGACCCAAAC -3′	5′-TGTACGTGGCTACATGGACCT -3′
*Pparg2*	Peroxisome proliferator activated receptor gramma 2	5′-TGTCGGTTTCAGAAGTGCCTTG-3′	5′-TTCAGCTGGTCGATATCACTGGAG-3′
*Ppargc1a*	Peroxisome proliferative activated receptor, gamma, coactivator 1 alpha	5′-ACAGCTTTCTGGGTGGATT-3′	5′-TGAGGACCGCTAGCAAGTTT-3′
*Ptgs1*	Prostaglandin-endoperoxide synthase 1	5′-GTGCTGGGGCAGTGCTGGAG-3′	5′-TGGGGCCTGAGTAGCCCGTG-3′
*Slc2a1*	Solute carrier family 2 (facilitated glucose transporter), member 1	5′-CCGCTTCCTGCTCATCAATC-3′	5′-CGACCCTCTTCTTTCATCTCC-3′
*Slc2a4*	Solute carrier family 2 (facilitated glucose transporter), member 4	5′-GTGACTGGAACACTGGTCCTA-3′	5′-CCAGCCAGTTGCATTGTAG-3′
*Tfam*	Transcription factor A mitochondrial	5′-GAGGCCAGTGTGAACCAGTG -3′	5′-GTAGTGCCTGCTGCTCCTGA -3′
*Ucp1*	Uncoupling protein 1	5′-GGCATTCAGAGGCAAATCAGCT-3′	5′-CAATGAACACTGCCACACCTC-3′

**Table 3:** Percentage yield of CQ-H fractions.

Fractions	Weight (g)	% Yield
1H	1.6752	0.28
2H	0.7629	0.13
3H	0.2637	0.04
4H	0.0830	0.01
5H	0.7585	0.13
6H	0.3403	0.06
7H	0.2472	0.04
8H	0.5413	0.09
9H	0.0840	0.01
10H	0.1143	0.02
11H	0.0864	0.01
12H	0.2380	0.04
13H	0.2762	0.05
14H	0.8879	0.15
15H	0.2510	0.04
16H	0.3697	0.06
17H	1.8605	0.31
18H	0.4872	0.08
19H	0.0799	0.01
20H	0.7666	0.13
21H	0.1261	0.02
22H	0.0888	0.01
23H	0.0729	0.01
24H	0.0503	0.01
25H	0.0700	0.01
26H	0.1846	0.03
27H	0.2121	0.04
28H	0.2961	0.05
29H	1.4841	0.25
30H	0.2945	0.05

**Table 4:** ^1^H and ^13^C NMR chemical shifts of Lu-CQ compared to those reported in the literature.

Position	Lupenone [21]		Lu-CQ	
	*δ*_H_ (ppm) (CDCl_3_, 300 MHz)	*δ*_C_ (ppm) (CDCl_3_, 75 MHz)	*δ*_H_ (ppm) (CDCl_3_, 300 MHz)	*δ*_C_ (ppm) (CDCl_3_, 75 MHz)
1	-	34.15	-	34.14
2	-	39.58	-	39.61
3	-	218.32	-	218.18
4	-	47.3	-	47.32
5	-	54.87	-	54.91
6	-	19.65	-	19.67
7	-	33.52	-	33.56
8	-	40.74	-	40.77
9	-	49.75	-	49.78
10	-	36.85	-	36.87
11	-	21.43	-	21.46
12	-	25.09	-	25.14
13	-	38.12	-	38.16
14	-	42.97	-	42.98
15	-	27.39	-	27.42
16	-	35.48	-	35.51
17	-	42.87	-	42.88
18	-	48.19	-	48.23
19	-	47.93	-	47.95
20	-	150.89	-	150.84
21	-	29.81	-	29.82
22	-	39.94	-	39.97
23	1.03 (3H, s, H-23)	26.6	1.03 (3H, s, H-23)	26.64
24	1.07 (6H, s, H-24)	21.02	1.07 (6H, s, H-24)	21.03
25	0.93 (3H, s, H-25)	15.96	0.93 (3H, s, H-25)	15.97
26	1.07 (6H, s, H-26)	15.75	1.07 (6H, s, H-26)	15.78
27	0.96 (3H, s, H-27)	14.45	0.96 (3H, s, H-27)	14.47
28	0.80 (3H, s, H-28)	17.99	0.80 (3H, s, H-28)	18.01
29	4.70 (1H, brs, H-29a), 4.58 (1H, brs, H-29b)	109.38	4.69 (1H, d, *J* = 2.1 Hz, H-29a), 4.57 (1H, d, *J* = 0.84 Hz, H-29b)	109.40
30	1.68 (3H, brs, H-30)	19.19	1.69 (3H, brs, H-30)	19.31

## Data Availability

All data used to support the findings of this study are available from the corresponding author upon request.

## Supplementary Materials

The following supporting information can be downloaded at: https://www.mdpi.com/article/10.3390/life13081724/s1, Figure S1: Standard curve of glucose content measured with an Accu-Chek blood glucometer; Figure S2: Microscopic images of 3T3-L1 cells at the undifferentiated state in complete growth media (CGM) and the adipogenic differentiated stage in adipogenic differentiation media (ADM) on day 8. (A) Live imaging of cells shows a difference in cell morphology, with the presence of lipid droplets only in the ADM condition. (B) Oil Red O staining confirms the induction of lipid droplet formation by ADM at low and high magnification. Figure S3: Cellular effects of CQ on mitochondrial density in 3T3-L1 cells. Cells were treated with Lu-CQ and 2H at concentration of 80 µg/mL. (A) Mitotracker staining showed mitochondrial distribution in red fluorescence. (B) Mean CTCF values. (C) Mean CTCF values of the mitochondrial density for each cell type. Values are presented as mean ± standard error. Statistically significant ** *p* < 0.01; Table S1: Gene expression of adipogenic marker with 18s normalization.
