# Supplementary material for: Lupenone-Rich Fraction Derived from Cissus quadrangularis L. Suppresses Lipid Accumulation in 3T3-L1 Adipocytes
Source: Life (Basel). 2023 Aug 11;13(8):1724. doi: 10.3390/life13081724 (PMC10455188; doi:10.3390/life13081724)
Supplement: Supplementary file 1 [file life-13-01724-s001.zip › life-2478537-supplementary.pdf]

Supplementary Data

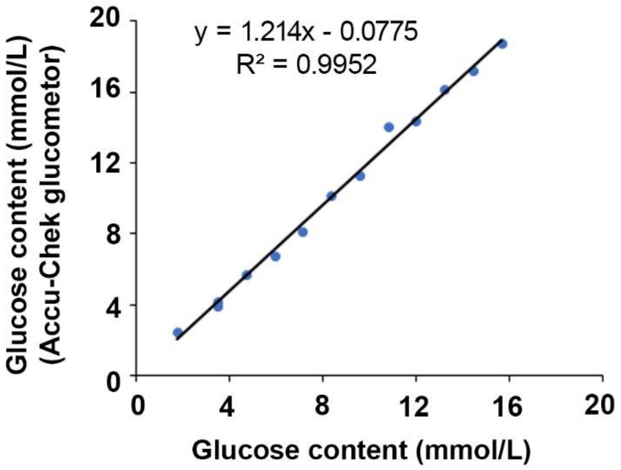

Figure S1. Standard curve of glucose content measured with an Accu-Chek blood glucometer.

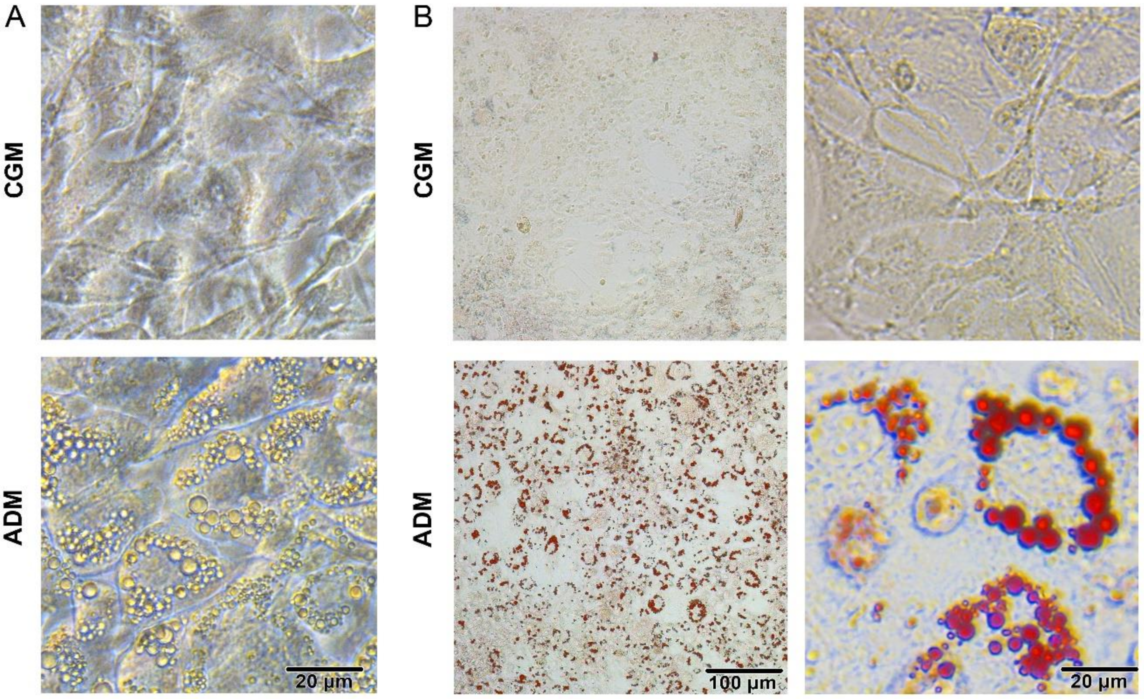

Figure S2: Microscopic images of 3T3-L1 cells at the undifferentiated state in complete growth media (CGM) and the adipogenic differentiated stage in adipogenic differentiation media (ADM) on day 8. (A) Live imaging of cells shows a difference in cell morphology, with the presence of lipid droplets only in the ADM condition. (B) Oil Red O staining confirms the induction of lipid droplet formation by ADM at low and high magnification.

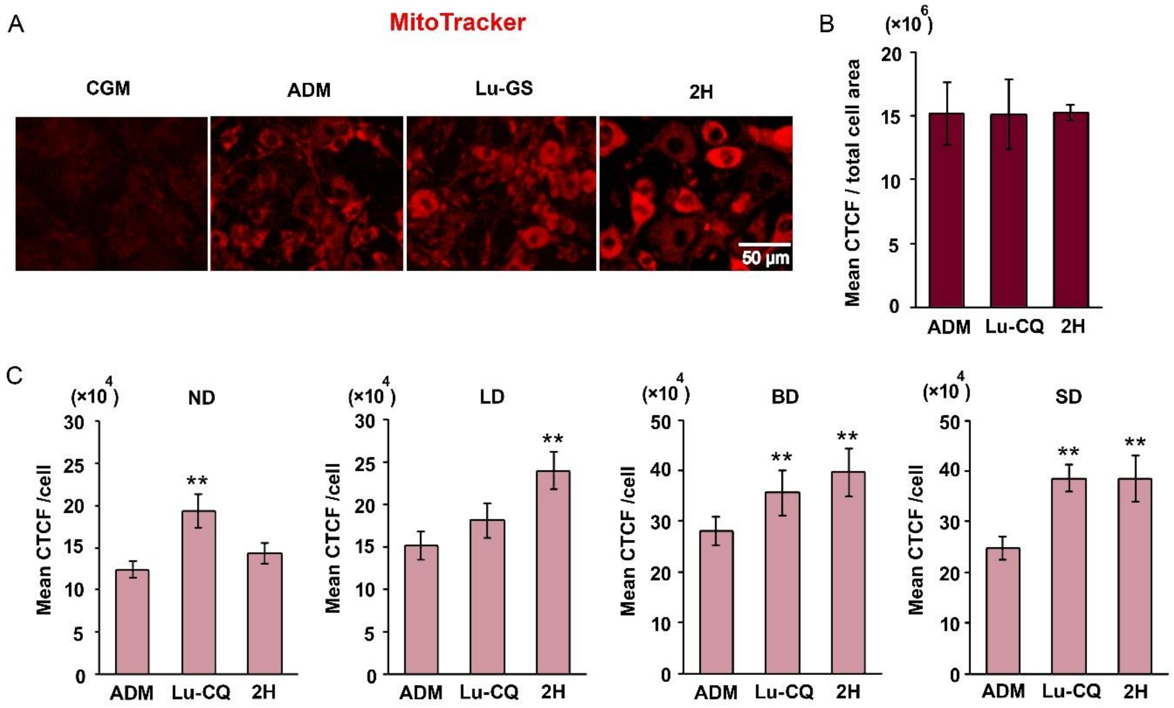

Figure S3. Cellular effects of CQ on mitochondrial density in 3T3-L1 cells. Cells were treated with Lu-CQ and 2H at concentration of 80 μg/mL. (A) Mitotracker staining showed mitochondrial distribution in red fluorescence. (B) Mean CTCF values. (C) Mean CTCF values of the mitochondrial density for each cell type. Values are presented as mean ± standard error. Statistically significant $^{**}p < 0.01$.

Table S1. Gene expression of adipogenic marker with 18s normalization

Gene symbol	Gene name	Threshold cycles value in CGM	Fold increase by ADM
18S	18S ribosomal RNA	7.96	-
Adipoq	Adiponectin	24.97	549.04
Cebpb	CCAAT/enhancer-binding protein beta	20.92	6.28
Pparg2	Peroxisome proliferator- activated receptor gramma 2	20.90	2.61
